# Antibiotic delivery evaluation against *Mycobacterium fortuitum* using nanofluids containing carbon nanotubes

**DOI:** 10.1186/s12866-022-02523-z

**Published:** 2022-04-11

**Authors:** Hamid Naderi Pargami, Seyed Davar Siadat, Vahid Amiri, Mojgan Sheikhpour

**Affiliations:** 1 Department of Biology, Faculty of Life Sciences, Danesh Alborz University, Abyek, Iran; 2 Department of Mycobacteriology and Pulmonary Research, Pasteur Institute of Iran, Tehran, Iran; 3 Microbiology Research Center, Pasteur Institute of Iran, Tehran, Iran

**Keywords:** *Mycobacterium fortuitum*, Functionalized multi-walled carbon nanotubes, Nanofluid, Antibiotic resistance

## Abstract

**Background:**

*Mycobacterium fortuitum (M. fortuitum)* is a bacterium, which can cause infections in many anatomical regions of the body, including the skin, lymph nodes, and joints. This bacterium, which belongs to a group of bacteria known as nontuberculous mycobacteria, is regarded as an important nosocomial pathogen worldwide owing to its increasing antibiotic resistance. Recently, the antimicrobial effects of carbon nanotubes have been reported in numerous studies. These nanotubes can be very useful in drug delivery; besides, they exhibit unique properties against multidrug-resistant bacterial infections. This study aimed to investigate the antimicrobial effects of carboxyl-functionalized multi-walled carbon nanotubes (MWCNT-COOH) to reduce antibiotic resistance.

**Methods:**

In this study, antibacterial effects of nanofluids containing functionalized MWCNTs at initial concentration of 2 mg/mL and serial dilutions of 54, 28.5, 14.25, 7.12, 3.5 µg/mL, antibiotics alone and combination of nanofluids with antibiotics were investigated.

Standard and resistant strains of *M. fortuitum* were obtained from the microbial bank of the Department of Mycobacteriology and Pulmonary Research, Pasteur Institute of Iran.

**Results:**

It was observed that nanofluid containing MWCNT-COOH can exert antimicrobial effects on *M. fortuitum* and significantly reduce bacterial resistance to antibiotics including kanamycin and streptomycin. In the presence of antibiotics and nanofluids containing MWCNT-COOH at a dose of 28.5 µg/mL, no growth was observed.

**Conclusion:**

One of the main antimicrobial mechanisms of MWCNT-COOH is penetration into the bacterial cell wall. In this study, by using the nanofluid containing MWCNT-COOH with increased stability, the antibiotic resistance of *M. fortuitum* was significantly reduced at lower dilutions compared to the antibiotic alone.

**Supplementary Information:**

The online version contains supplementary material available at 10.1186/s12866-022-02523-z.

## Introduction

Non-tuberculous mycobacterial (NTM) is a challenging infection that has spread for a number of reasons, including antibiotic resistance. They are organisms found everywhere in the environment around the world and are resistant to extreme heat and pH and many disinfectants and antibiotics due to their thick fat cell wall [1]. *Mycobacterium fortuitum* was first described in 1938 by Costa Cruz who isolated it from the pus of an abscess in a woman who had received injections of a vitamin preparation This pathogen is widely distributed in soil and water worldwide [2]. Generally, the NTM can be classified depending on their growth rate. Accordingly, *M. fortuitum* is known as a rapidly growing mycobacterium (RGM) [3].

*M. fortuitum* infection is recognized as a nosocomial infection, which can affect many areas of the body, including the skin [4], lymph nodes [5], lungs [6], and bones (osteomyelitis), especially after a catheterization surgery [7, 8]. The *M. fortuitum* group consists of about 15 fast-growing NTM species. These globally distributed microorganisms can cause disease in humans and animals [9]. The presence of *M. fortuitum* in the respiratory tract has been reported mainly as a simple or transient infection. However, *M. fortuitum*-induced lung infection remains rare and generally occurs in patients with gastroesophageal reflux disease or in elderly patients with chronic cough [10, 11]. The antibacterial resistance of nosocomial infections has become a global health concern [12]. Due to differences in susceptibility between Mycobacterium species with rapid growth and even within species, susceptibility testing should be performed on all clinically important isolates as well as isolates that have improved after treatment failure or recurrence. Mycobacterium fast-growing antimicrobial susceptibility testing differs from other NTMs. Most drugs are different, although the methods are similar to those used to test for other bacteria [13, 14]. Infections caused by *M. fortuitum* isolates also require long-term antibiotic treatment due to their high resistance to a variety of antibiotics and disinfectants [15]. Previous studies have shown that *M. fortuitum* strains are susceptible to fluoroquinolones and amikacins, while they are resistant to macrolides; therefore, understanding the mechanisms of drug resistance and effective treatments for *M. fortuitum* infections are essential [16, 17]. Currently, the use of the combination of antibiotics has been able to control infections caused by *M. fortuitum* to some extent [18].

Over the past few years, there has been a growing interest in using nanoparticles in various biomedical applications such as targeted drug delivery, bioimaging, and biosensors [19]. Various nanoparticles such as carbon nanotubes (CNTs) have been used for antibiotic resistance. CNTs discovered by Iijima in 1991 have enhanced technological advances in nanotechnology [20]. Due to their high surface-to-volume ratio and unique physical and chemical properties, particular attention has been paid to the antibacterial properties of nanoparticles [21]. CNTs are one of the most important types of nanoparticles.

Recently, cancer diagnosis and treatment, as well as antibiotic resistance, have become important research topics [22, 23]. The CNTs can exist into two categories depending upon the number of layers: single-walled carbon nanotubes (SWCNTs) and multi-walled carbon nanotubes (MWCNTs). The antibiotic properties of MWCNTs have been reported in previous studies [24, 25]. It seems that the functionalization of MWCNTs with a carboxyl agent (MWCNT-COOH) can improve their antimicrobial properties [26]. The present study aimed to investigate the antimicrobial effects of nanofluid containing MWCNTs-COOH and to examine their simultaneous use with antibiotics, including kanamycin and streptomycin, against *M. fortuitum* to reduce its antibiotic resistance.

## Materials and methods

### Preparation of multi-wall carbon nanotube (MWCNTs) based aqueous nanofluid

The MWCNTs (US Research Nanomaterials, Inc., USA) were used in the present study. For this purpose, 0.2 g of MWCNT-COOH powder, 6 mL of 96% ethanol, and 0.06 g of Arabic gum were added to 100 mL of deionized water, and the mixture was stirred for 20 min [27]. Next, the solution was placed in an ice bucket. The suspension was placed in an ice bucket to the ultrasonic device (Ultrasonic Homogenizer 400 w, 200 kHz), with the power of 200 W for 20 min.

### Bacterial strains and culture conditions

Two *M. fortuitum* strains (resistant and ATCC 6841) were provided by the microbial bank of the Department of Mycobacteriology and Pulmonary Research of Pasteur Institute of Iran. Resistant strains were isolated from clinical specimens. The ATCC and resistant strains were cultured in a Lowenstein-Jensen (LJ) medium and incubated at 37 °C [28]; bacterial growth was monitored at 4 weeks. Antibiotic susceptibility of bacterial strains was determined by antibiogram method according to CLSI guidelines. Ofloxacin (35 µg/mL), streptomycin (32 µg/mL), kanamycin 30 (µg/mL), ethambutol (256 µg/mL), rifampicin (32 µg/mL), and isoniazid (16 µg/mL) were used in this study to treat pulmonary diseases and control M. fortuitum [29]. At this stage, a 0.5-McFarland microbial suspension was prepared from bacterial strains, and then, antibiotics were added to the LJ medium. After a week, the final results were observed.

### Microbial testing

First, a 0.5-McFarland microbial suspension was purified from bacteria, and then, different dilutions (54, 28.5, 14.25, 7.12, 3.5 µg/mL) of the nanofluid at a concentration of 2 mg/mL were prepared and added to each strain; the tests were performed twice for each sample. The LJ medium was incubated at 37 °C during the first week of bacterial growth. Besides, the simultaneous effects of the nanofluid and antibiotics on the ATCC and resistant strains were examined. Different concentrations of the nanofluid were used in this study, with an initial concentration of 2 mg/mL. The LJ culture media, containing streptomycin and kanamycin, were prepared for both ATCC strains and pathogens.

### Molecular detection

The *M. fortuitum* RNAs were extracted using Triazole (GeneAll RiboEx) and then, stored at -70 °C until further cDNA synthesis. Following RNA extraction, cDNA synthesis was carried out using a Takara cDNA synthesis kit (Takara Bio Inc., Japan). Each cDNA sample was amplified using specific primers, Gene expression level was comparable to 16S rRNA of housekeeping [30] (Table 1).

**Table 1:** Primer sequences used in the study

Locus	Primer	Product size
blaF_ forward	5’_ CCTGTTGGAAGACTGGATG_3’	112 bp
blaF_ reverse	5’_ GTTGGTGCTGCCGTAATC_3’	
Aph(3″)_IC _ forward	5’_ CTGGCGGTGTGGGGTATT_3’	103 bp
Aph(3″)_IC _ reverse	5’_ CGTCGGAGTTCCTGAAGA_3’	
16 s rRNA _ forward	5’_ GACTGCCAGACACACTATTGG_3’	172 bp
16 s rRNA _ reverse	5’_ GTGAGACCACACGATTCTGC_3’	172 bp

In this study, expression level of *aph(3'')-Ic* and *Blaf* genes involved in a aminoglycoside O-phosphotransferase and beta-lactamase pathways were examined. Moreover, real-time polymerase chain reaction (PCR) was performed using a QIAGEN apparatus under the following conditions in 40 cycles: at 95 °C for five seconds; at 60 °C for 20 s; and at 72 °C for 30 s. Amplification was also performed using an SYBR Premix Ex Taq™ II PCR Master Mix (Takara Bio Inc., Japan). The gene expression data were analyzed using the 2^−ΔCT^ and 2^−ΔΔCT^ methods after normalization.

## Results

### Microbial testing results

**Fig. 1:**
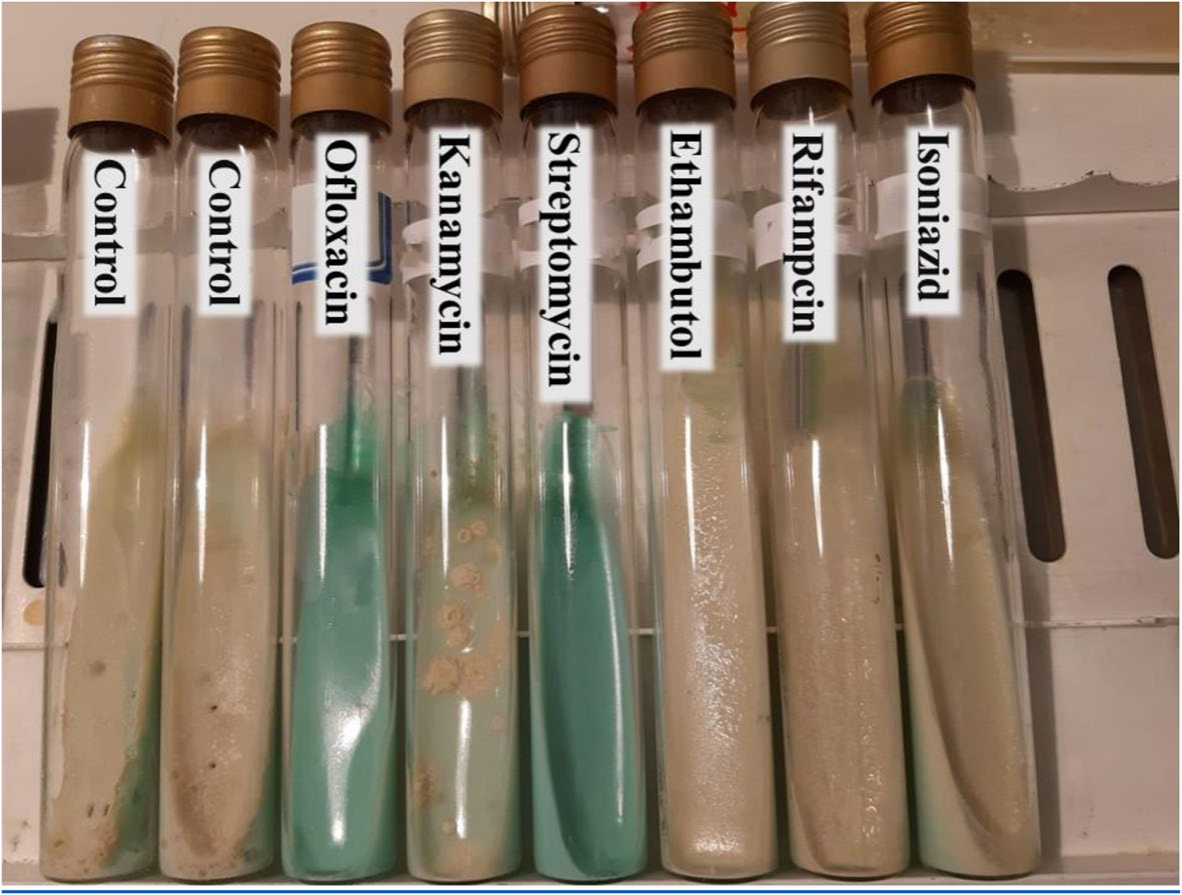
Results of antibiogram testing in ATCC strain. ATCC strains show sensitivity to antibiotics ofloxacin and streptomycin

**Fig. 2:**
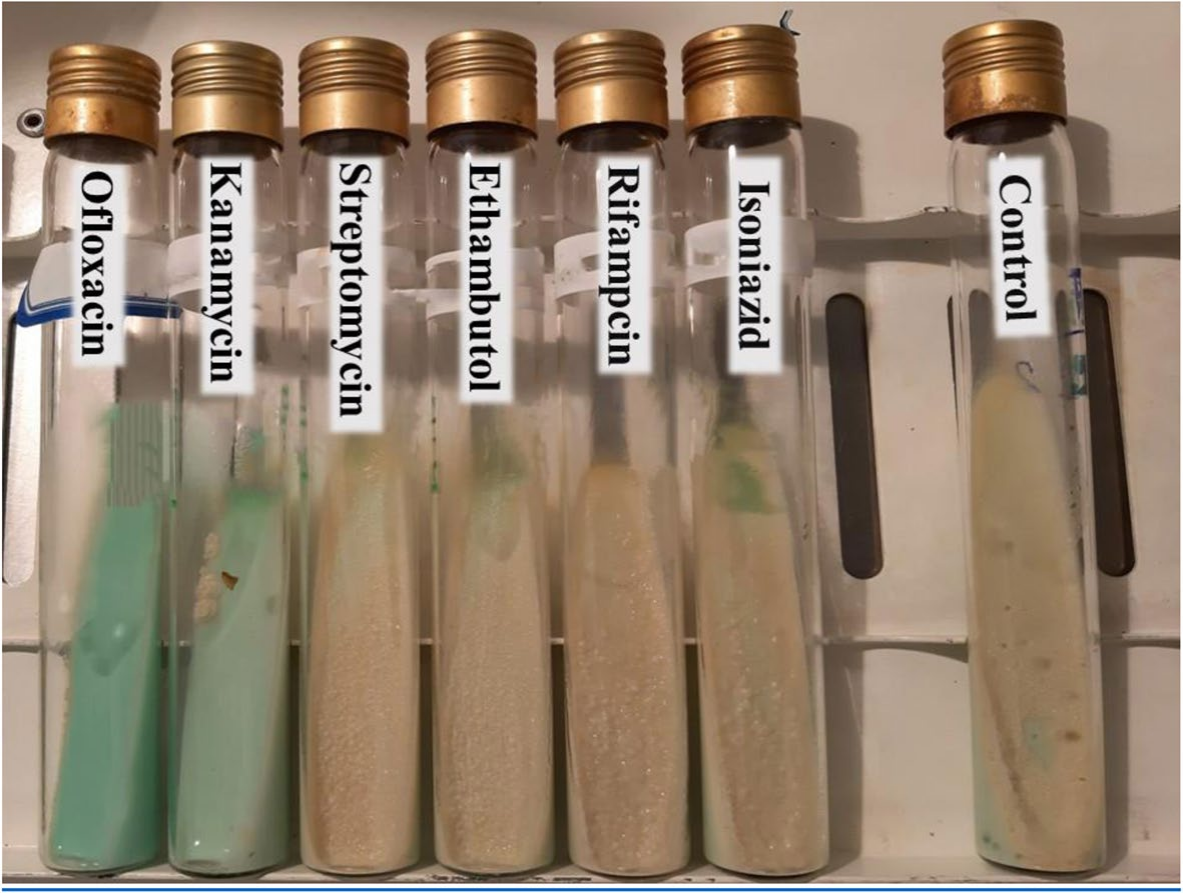
Results of antibiogram testing in pathogen strain. Resistance strains show sensitivity to antibiotics ofloxacin and kanamycin

The antibiograms showed that the ATCC strain of *M. fortuitum* was susceptible to ofloxacin and streptomycin (Fig. 1), while the resistant strain was susceptible to ofloxacin and kanamycin (Fig. 2).

The bacterial culture results were studied, and the antibacterial efficacy of the nanofluid was examined after two weeks. The examination of the effects of the nanofluid on the ATCC strain showed no bacterial growth at a dilution of (28.5 µg/mL). In the resistant strain, no growth was observed at a dilution of (54 µg/mL). Moreover, analysis of the simultaneous effects of the nanofluid and antibiotics on bacteria showed no bacterial growth in the ATCC strain at a dilution of (14.25 µg/mL) with kanamycin, while in the resistant strain, no growth was observed at a dilution of (28.5 µg/mL) using streptomycin (Table 2), (Figures S1 and S2).

**Table 2:** Summary of the effects of drugs and carbon nanotubes in this study

Treatments	Strains	
	***M. fortuitum***	
	***The dilution that inhibited bacterial growth (µg/mL)***	
	**ATCC**	**Resistance**
**MWCNT-COOH**	28.5	54
**MWCNT-COOH + Kanamycin**	14.25	28.5
**MWCNT-COOH + Streptomycin**	14.25	28.5

### Results of statistical analysis

**Fig. 3:**
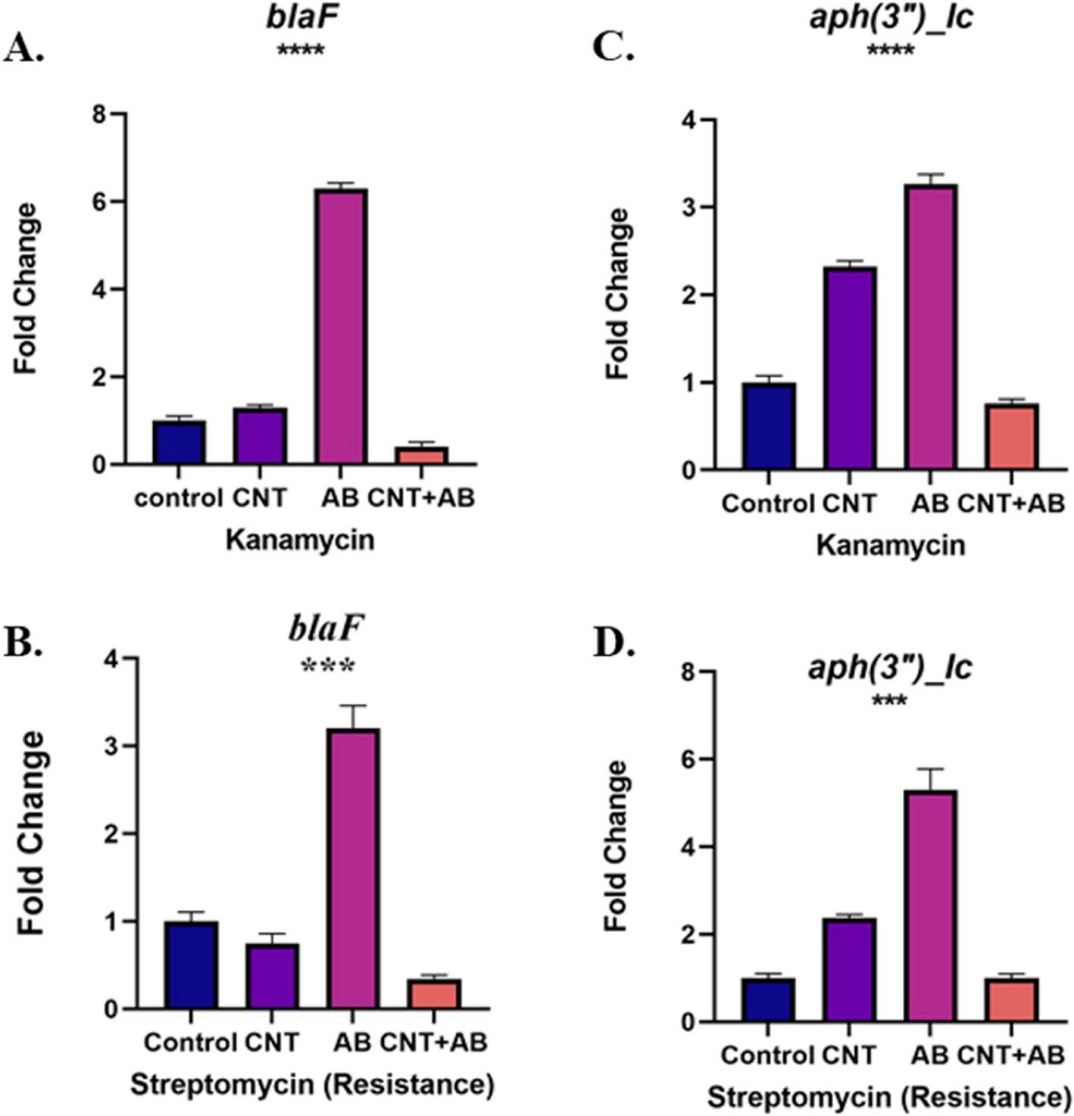
**A** *Bla* gene expression level in *ATCC* strain. **B** *Bla* gene expression level in resistance strain. **C** Aph(3″) gene expression level in ATCC strain. **D** Aph(3″) gene expression level in resistance strain

Statistical analysis of *aph(3'')-Ic* gene expression and blaF was performed using one-way ANOVA to compare the results of the group by GraphPad Prism 8 (San Diego, California, USA).A P value less than 0.05 was considered significant. The analysis of gene expression showed that the expression of *aph(3'')-Ic* and blaF genes decreased more significantly following exposure to a combination of the nanofluid and antibiotics, compared to the nanofluid or antibiotic alone; the expression levels of the genes were comparable to that of 16S rRNA housekeeping gene (Fig. 3).

## Conclusions and implications for future research

*M. fortuitum* is one of the most well-known NTMs that can cause various infections, including lung and wound infections [11]. Results of this study showed that nanofluids containing functionalized (carboxyl), carbon nanotubes have significant antibacterial effects and their effects were examined alone or in combination with common antibiotics, such as kanamycin and streptomycin. This nanofluid contained carbon nanotubes with superior characteristics, such as increased penetration into bacterial membranes, increased efficiency at lower concentrations compared to common therapeutic doses, and reduced bacterial resistance to common antibiotics.

In a study, the antibacterial performance of silver-functionalized and non-functionalized carbon nanotubes was compared. It was found that silver-functionalized carbon nanotubes exerted antibacterial effects on *Escherichia coli* [31]. Another study in 2020 evaluated the effects of isoniazid-functionalized MWCNTs on *M. tuberculosis*. Their results showed that isoniazid-functionalized MWCNTs exerted antimicrobial effects at lower concentrations compared to the usual doses of antibiotics [32]. In a recent study. The antimicrobial effects of nanofluid containing functional carbon nanotubes on *Acinetobacter baumannii* were studied. This study showed that nanofluids containing nanotubes with carbon results could have significant antibacterial effects on *Acinetobacter baumannii* [33]. In another study, the molecular effects of combination therapy with antibiotics and carbon nanotubes containing nanofluids on *Klebsiella pneumoniae* were performed. The results of this study showed that the combination of antibiotics with carbon nanotubes could significantly inhibit bacterial growth [34]. Using a variety of antibiotics to treat infections caused by *Mycobacterium fortitum* can have side effects. Nowadays, it is possible to reduce the side effects of antibiotics such as the metabolic side effects by using nano drug delivery systems.

The present study showed that the efficacy of nanofluids containing MWCNTs, functionalized with carboxylic acid, differed from that of nanofluids containing non-functionalized MWCNTs. This difference appears to have changed following bacterial exposure to the nanofluid, which might be related to decreased bacterial growth due to the binding of the nanofluid containing carbon nanotubes (functionalized with carboxylic acid) to the bacterial membrane, thereby eliminating the membrane integrity. On the other hand, based on molecular studies and analysis of *bla* and *aph(3″)-IC* gene expression, the number of bacteria significantly reduced in the presence of CNTs. However, further studies are needed to examine this method (i.e., the combination of carbon nanotubes with antibiotics in the form of nano-drugs) and obtain better results. Also, further cellular and molecular studies are recommended to identify the mechanisms of antibacterial effects.

## Supplementary Information

**Additional file 1.**

## Data Availability

All data generated or analyzed during this study are included in this published article [and its supplementary information files].
