# Supplementary material for: Antibiotic delivery evaluation against Mycobacterium fortuitum using nanofluids containing carbon nanotubes
Source: BMC Microbiol. 2022 Apr 11;22:96. doi: 10.1186/s12866-022-02523-z (PMC8996581; doi:10.1186/s12866-022-02523-z)
Supplement: Supplementary file 1 — Additional file 1. [file 12866_2022_2523_MOESM1_ESM.docx]

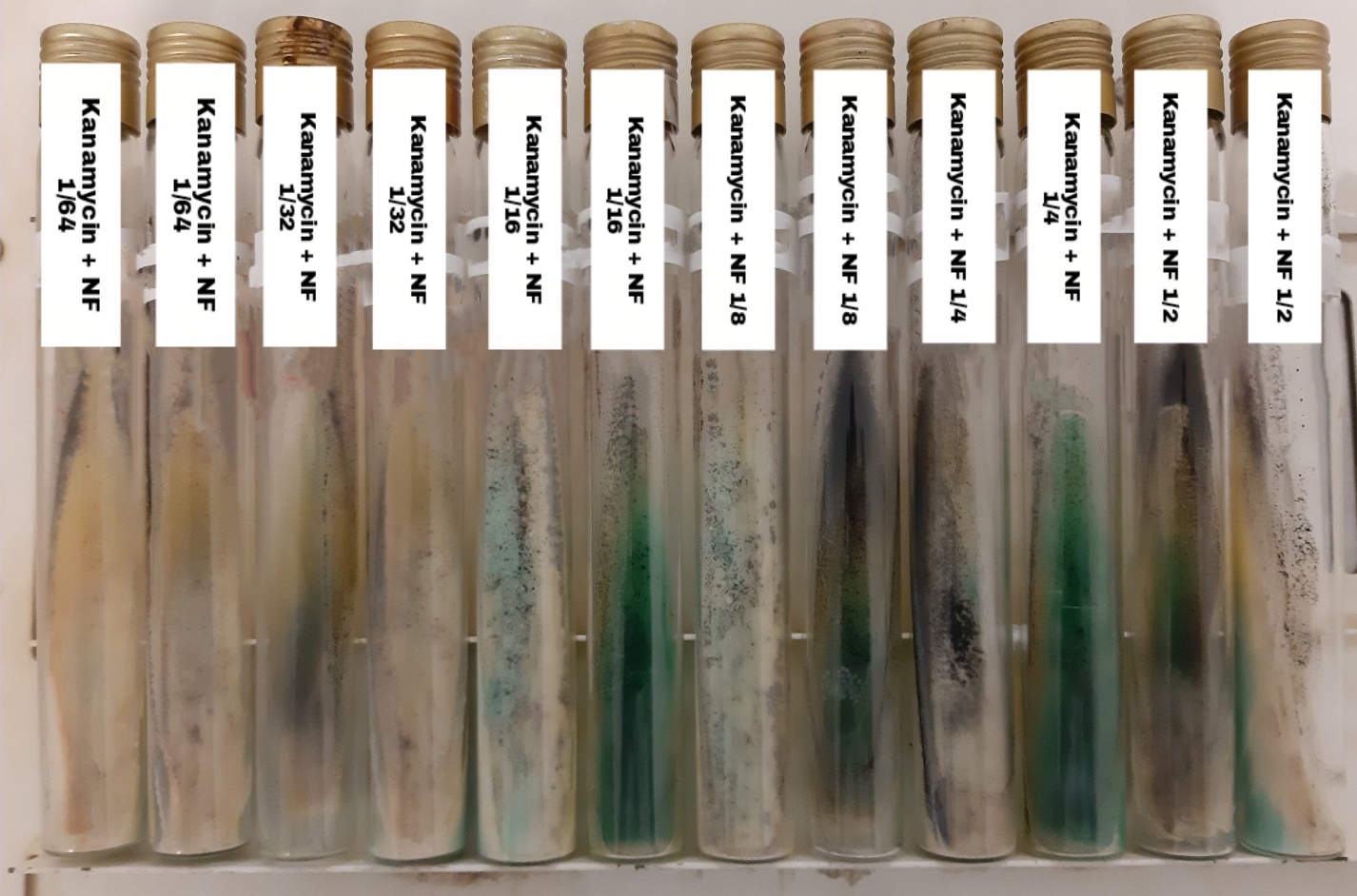


**S1. Results of antibiotic and nanofluid efficacy simultaneously.** No growth was observed at a dilution of 1:4 (28 **µg/mL)**


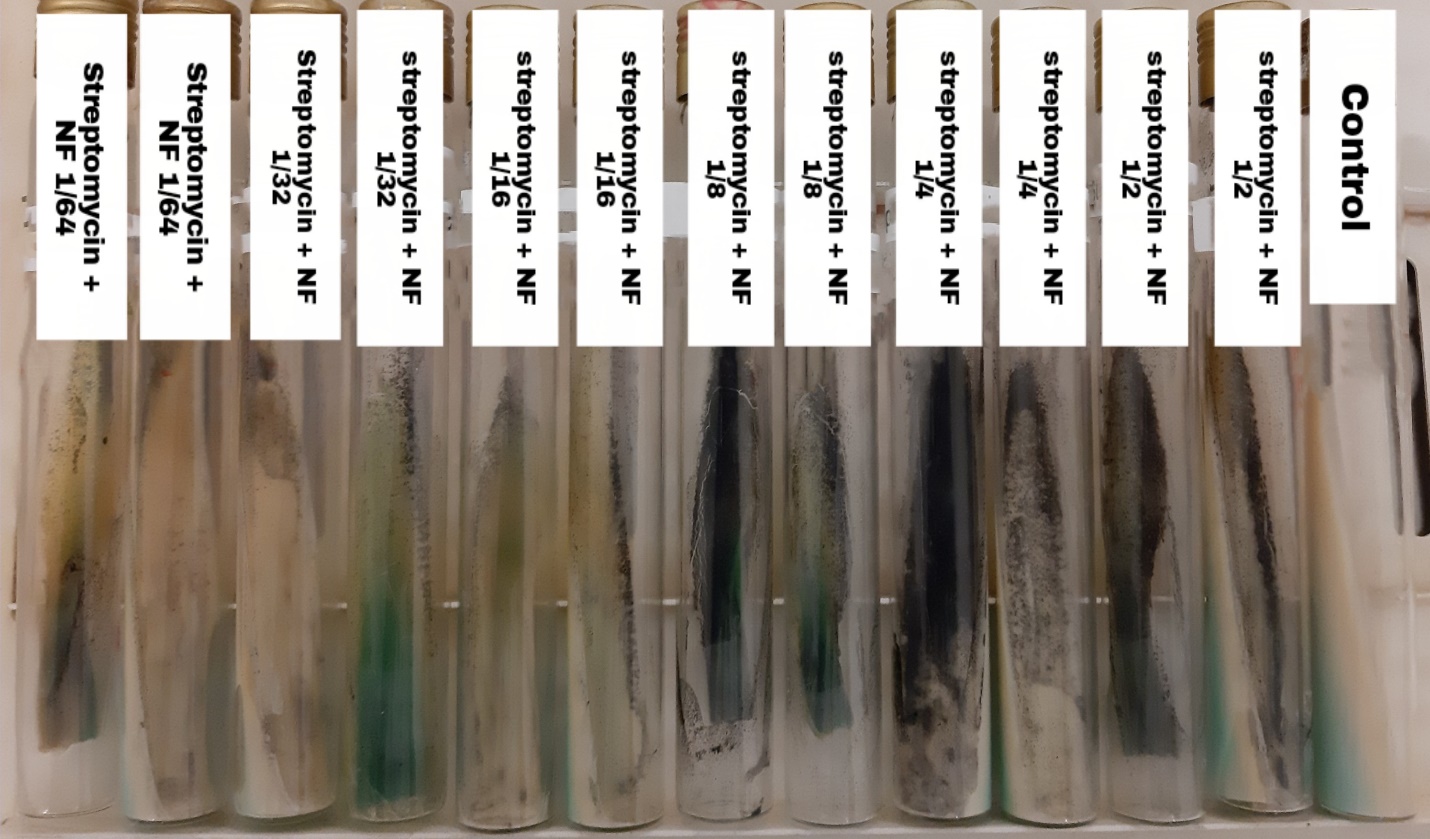


**S2. Results of antibiotic and nanofluid efficacy simultaneously.** No growth was observed at a dilution of 1:4 (28 **µg/mL)**


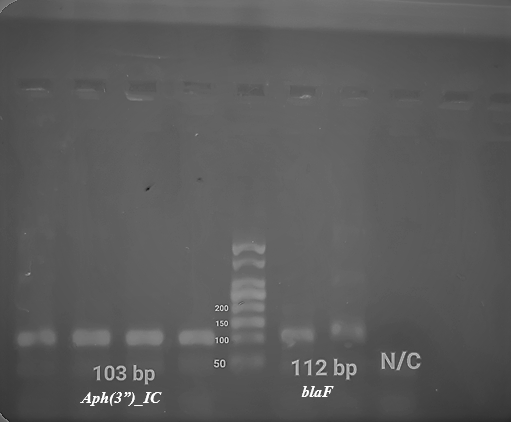


**S3. Agarose gel electrophoresis for amplified Aph(3”)_Ic and BlaF gene.**
